# Age- and Gender-Based Differences in Nest-Building Behavior and Learning and Memory Performance Measured Using a Radial Six-Armed Water Maze in C57BL/6 Mice

**DOI:** 10.1155/2018/8728415

**Published:** 2018-05-09

**Authors:** Xiang-Dong Xiong, Wei-Dong Xiong, Shang-Shen Xiong, Gui-Hai Chen

**Affiliations:** ^1^Department of Neurology, The First Affiliated Hospital of Anhui Medical University, Hefei, 230022 Anhui, China; ^2^Lu'an Affiliated Hospital of Anhui Medical University, People's Hospital of Lu'an City, Lu'an, 237005 Anhui, China; ^3^Hefei National Level High and New Technology Development Zone, Hefei, 230088 Anhui, China; ^4^Department of Neurology, The Affiliated Chaohu Hospital of Anhui Medical University, Chaohu, 238000 Anhui, China

## Abstract

**Background:**

Understanding age-based and gender-based behavioral changes is becoming more important as a greater percentage of people lives longer worldwide. In this study, a C57BL/6 mouse animal model was used to study age-based and gender-based behavioral differences using nest building and radial six-armed water maze (RAWM) testing.

**Methods:**

In C57BL/6 mice, nest-building behavior was recorded as nesting scores, while spatial learning and memory behaviors were assessed using RAWM platform search errors and latencies.

**Results:**

In the nest-building test, nest building significantly declined in nineteen 25-month-old mice compared to that of twenty-three 7-month-old mice. Meanwhile, nest building in 25-month-old mice was lower for eight male mice than for eleven female mice, while no significant gender differences were observed in nest building of 7-month-old mice. RAWM performance also declined in aged versus nonaged adult mice, while no significant gender differences were observed in average RAWM performance regardless of age.

**Conclusions:**

In adult C57BL/6 mice, nest building is a sensitive indicator for detecting both age- and gender-based behavioral declines, while RAWM performance, an assessment of spatial learning and memory behaviors, is not sensitive to gender but significantly declines with aging. Therefore, for a C57BL/6 mouse model of aging, both nest building and RAWM should be useful to further study mechanisms involved in behavioral decline with aging.

## 1. Introduction

Understanding age-based and gender-based behavioral changes is becoming more important as the elderly population grows. Memory behavioral impairment with advancing years not only reduces the quality of life for the elderly but also increases the costs of healthcare for society. In order to understand the mechanisms that give rise to behavioral decline with aging and gender-based behavioral differences, animal models are needed to unravel the complex changes that disrupt brain function of the aging brain.

In mice, although species' typical behaviors include nest building [1, 2], age- and gender-related nest-building behavioral differences in adult mice have not been reported. Similar age-based and gender-based spatial and memory behavioral studies in radial six-armed water maze (RAWM) spatial cognitive test in adult mice have not yet been reported even though C57BL/6 mouse strain possesses advantages as an animal model. Aside from the fact that C57BL/6 is one of the most commonly used laboratory mouse strains, these mice are also excellent swimmers (useful in water maze tests) and have a short lifespan. It is well known that there are about 30,000 genes in mice similar to detected gene numbers of humans. The interest in C57BL/6 mice and other mouse strains is because of their frequent use in transgenic research. It is well known that there are gender- and age-based differences on cognitive tasks that require the use of spatial learning and memory tests in mice. The only novelty is the use of the radial six-armed water maze test. Moreover, aged C57BL/6 mice (25-month-old) have been shown to exhibit impaired spatial memory relative to nonaged adult mice (five-month-old, but not nonaged adult 17-month-old) using the Morris water maze (MWM) spatial cognitive test, but no significant gender differences were detected by MWM testing [3]. Thus, for the aforementioned reasons, C57BL/6 mice may serve as a preferred animal model to study human aging. Using this animal model, we tested the following: (1) nest-building behavior would be sensitive to both age and gender differences in adult C57BL/6 mice; (2) spatial memory behavior measured using RAWM would detect age- and gender-based spatial memory behavioral changes in adult C57BL/6 mice; (3) behavioral differences measured by nest building and RAWM would show similar trends for aged versus nonaged adult C57BL/6 mice. All of these initiatives were experimentally addressed, and results reported here should encourage future use of this animal model for research to investigate mechanisms of human memory decline with aging.

## 2. Materials and Methods

### 2.1. Animals and General Protocols

C57BL/6 mice were purchased from the Experimental Animal Center of the University of Science and Technology of China (Hefei) and were maintained as an inbred strain and bred under specific pathogen-free conditions. Mice were transferred to conventional housing conditions at two months of age, housed in groups of 4 to 6 same-gender mice per cage in 25.5 × 15 × 14 cm plastic cages with wood shavings. Mice were allowed free access to a standard rodent diet and tap water during a 12 h light-dark cycle, with lights turned on at 7 : 30 AM and off at 7 : 30 PM. Mice were housed at a constant temperature of 21-22°C and humidity of 55% (±5%). Mice were preselected for overall health and normal behavioral attributes, and animals with gross defects (tumors outside trunk, motor incapacitation, or overt blindness) were excluded from experimentation. A total of 42 C57BL/6 mice comprised two groups: twenty-three 7-month-old (nonaged adult) mice, including ten males and thirteen females, and nineteen 25-month-old (aged) mice, including eight males and eleven females. The body weight of aged mice was similar to that of nonaged adult mice. One hour prior to testing, the mice were transferred to the experimental room for acclimatization. The nest-building test was performed during the dark phase of the light-dark cycle, and remaining tests were performed during the light phase. The order of completed tests was as follows: days 1-2 nest building, day 3 MWM cued learning, and days 4–10 RAWM. The Ethics Committee of The First Affiliated Hospital of Anhui Medical University approved this study, and all experimental protocols were approved by the hospital's Institutional Review Committee using procedures in compliance with the National Institutes of Health Guide for Care and Use of Laboratory Animals.

### 2.2. Nest-Building Test and Nesting Scores

As previously described [1, 2], mice were individually housed overnight with food, water, and new sawdust bedding. Six pieces of 5 × 5 cm^2^ white paper (which mice use to make nests) were placed in each cage. After the overnight nest-building test, nests typically contained with a shallow crater of sawdust spread, surrounded by, or covered with shredded or whole paper. The mice were scored according to the following nesting score scale: 0 = no visible crater of sawdust and no paper; 1 = sawdust crater alone and no shredded paper; 2 = sawdust crater with shredded or whole paper gathered around and in the crater; 3 = sawdust crater with shredded or whole paper gathered around and in the crater forming a cup-shaped nest; 4 = shredded paper forming a ball-shaped nest covering the mouse.

### 2.3. Cued Learning Using a MWM

A circular black tank 150 cm in diameter and 30 cm in height [4–6] was used. The initial water temperature of 20-21°C [6] was decreased by 1-2°C in the MWM as compared to the temperature of 21-22°C in the RAWM in order to increase aversion to swimming and decrease floating tendency. The tank was situated in an identical orientation to that of the RAWM, with a distance of 75 cm from the tank wall to the white cloth curtain. Cued learning tests were used to evaluate swimming ability, visual acuity, nonspatial learning ability, and motor or sensorimotor function. A black escape platform with diameter 10 cm and height 24 cm was raised 1.0 cm above the water surface, and a 7 cm diameter plastic ball with black and white stripes was attached to the top of the platform with a short rod. The platform position was different for each trial, but the release point was identical for each of five consecutive trials performed on a single day. Latency in finding the visible platform was recorded for a maximum of 60 s. After finding the visible platform, a mouse was allowed to stay there for a 30 s rest. Mice that failed to find the visible platform were guided there and allowed to remain there for 30 s.

### 2.4. Apparatus and Procedure for RAWM Testing

As described previously [4, 5], a RAWM was constructed using a circular black water tank 100 cm in diameter and 21 cm in depth placed atop a 30 cm high steel rack and filled with 21-22°C water to a depth of 16 cm. A white cloth curtain surrounded the tank at a distance of 150 cm from the tank wall. Three black cardboard pieces, either a circle, triangle, or square (each 30 cm in height), were hung on the curtain equidistantly from the edges of the curtain, and these pieces served as spatial cues. Four 100 W evenly spaced floodlights were placed on the ground around the curtain. The tank contained six 30.5 × 19 × 21 cm^3^ swimming alleys which radiated out from the 40 cm diameter center area. A black escape platform (10 cm in diameter) was submerged 1.0 cm below the water surface and was placed at the peripheral end of one alley a distance of 4.5 cm from the walls. During testing, both the platform and the experimenter remained at a constant position for seven consecutive days. On each day, four consecutive acquisition trials (defined as RAWM learning phase) followed 30 minutes later by one memory retention trial (defined as RAWM memory phase) were completed. On the first day, each mouse was first placed on the submerged platform for 30 s. From trial 1 to trial 4, the mouse was individually released into the water from four random entry alleys (except from the alley containing the platform and its opposite alley, to avoid the effect of innate direction bias) [7]. In each trial, the mouse was able to find the submerged platform within 60 s and allowed to rest on the platform. After 30 s of rest, it was taken off the platform. Upon entering an incorrect alley with its entire body, or if the mouse failed to select an alley within 10 s, the mouse was gently placed at the start alley; thus, this attempt was defined as an error. After the acquisition trials were completed, the mouse was towel-dried and placed in the home cage under a 150 W floodlight for 30 minutes. The mouse was then returned to the maze to complete trial 5 with the start alley identical to trial 4. Latency was defined as the time from the mouse's entry into the water to the time of finding the submerged platform. Due to the daily changing of sequences of starting points for trials 1–4 and innate swimming direction bias, spatial learning performance of each mouse did not always improve from trial 1 to trial 4. The number of errors and latencies in the daily trials during the 1-to-4-day period was averaged for statistical analysis.

### 2.5. Statistical Analyses

All results were expressed as the mean ± standard error of the mean (mean ± SEM) values. The Kruskal-Wallis test was used to verify effects of age and gender for the nonparametric data of nest building. For RAWM, analysis of variance (ANOVA) was used, using the statistical design of an age × gender × testing day-mixed ANOVA, with age and gender serving as between-subject factors and testing day as a repeated measure. A value of *P* < 0.05 was considered statistically significant. All analyses were conducted using SPSS 16.0 for Windows.

## 3. Results

### 3.1. Nesting Scores

**Figure 1:**
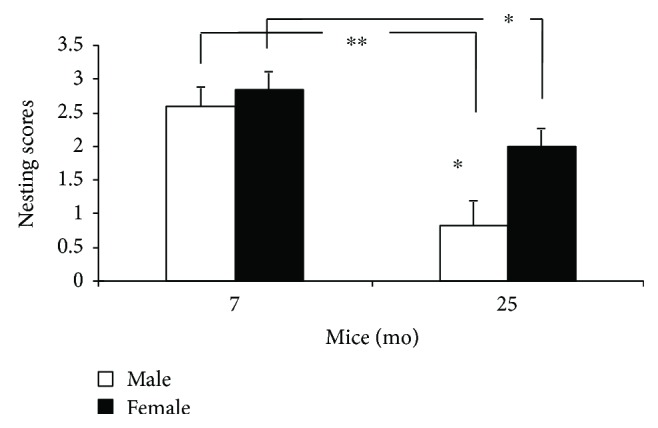
Nesting scores in 7- and 25-month-old C57BL/6 male and female mice (mean ± SEM). ^∗^*P* < 0.05; ^∗∗^*P* < 0.01.

For all C57BL/6 mice, nesting scores and nest building significantly declined in aged mice compared to results of nonaged adult mice [*x^2^*_(1,40)_ = 10.113, *P* = 0.001 (<0.01)]. Moreover, age had a significant effect on both male and female mice [*x^2^*_(1,16)_ = 7.155, ^∗∗^*P* = 0.007 (<0.01); *x^2^*_(1,22)_ = 4.345, ^∗^*P* = 0.037 (<0.05)] (Figure 1). Nesting scores significantly declined for aged male mice as compared to scores of aged females [*x^2^*_(1,15)_ = 4.770, ^∗^*P* = 0.029 (<0.05)] (Figure 1). However, no significant gender differences in nesting scores and nest building were observed for 7-month-old nonaged adult mice [*x^2^*_(1,21)_ = 0.217, *Ps* = 0.641 (>0.05)] (Figure 1).

### 3.2. Latencies in MWM Cued Learning

**Figure 2:**
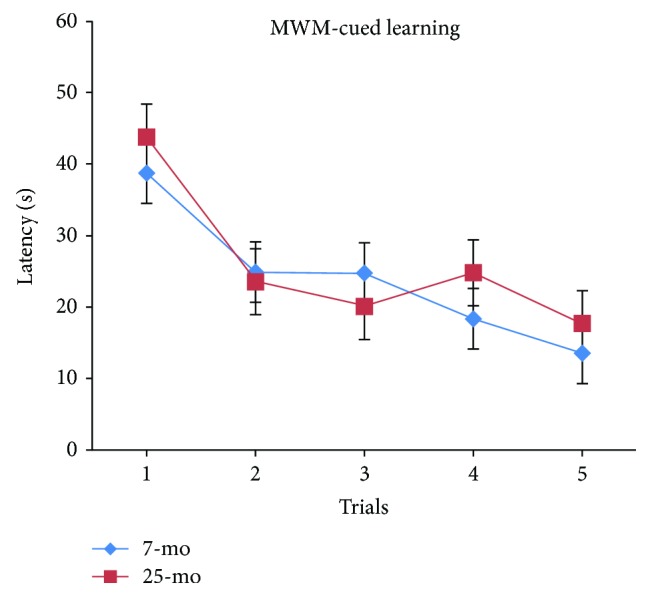
Latencies of 7- and 25-month-old C57BL/6 mice in cued learning of MWM (s, mean ± SEM).

**Figure 3:**
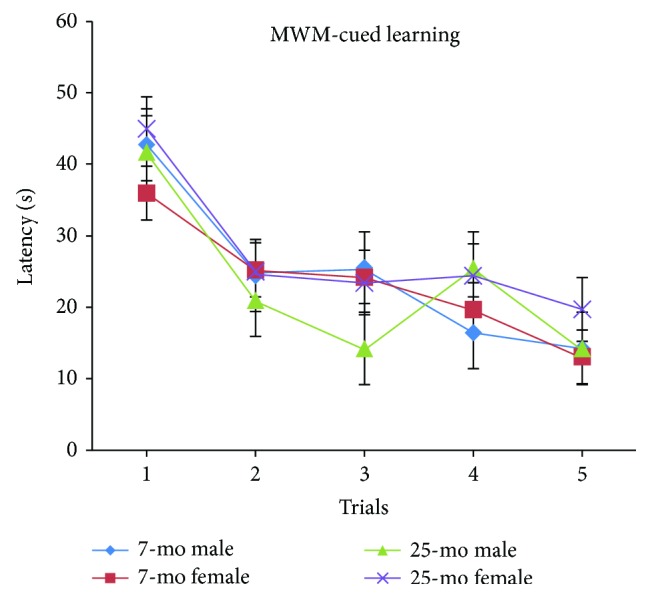
Latencies of 7- and 25-month-old C57BL/6 male and female mice in cued learning of MWM (s, mean ± SEM).

Latencies (time taken to find the visible platform after entering the water) were progressively and significantly decreased with higher trial number [*F*_(4,116)_ = 11.718, *P* < 0.001] (Figure 2). Latencies significantly decreased either from trial 2 onward or from trials 3 to 5 onward compared to latencies for trial 1 (*P* < 0.01 or *P* < 0.001). In general, during MWM cued learning latency, periods are known to depend on factors that include nonspatial learning ability, visual acuity, and motor or sensorimotor function. In this work, no significant differences were observed in latency between aged and nonaged adult C57BL/6 mice [*F*_(4,132)_ < 1, *Ps* > 0.05] (Figure 2) and no significant gender differences were observed in aged versus nonaged adult mice (*Ps* > 0.05) (Figure 3). By virtue of the constant swimming distance of 100 cm to the visible platform for each of the five consecutive trials, latencies could be reflected by variations in swimming speeds. However, with regard to swimming speeds during latency during MWM cued learning, no significant differences between aged and nonaged adult mice (*Ps* > 0.05) were observed and no significant gender-based differences were observed regardless of age (*Ps* > 0.05). Furthermore, no increased tendency to float was observed in aged mice versus nonaged mice during MWM cued learning.

### 3.3. Errors and Latencies during the Learning Phase of RAWM

**Figure 4:**
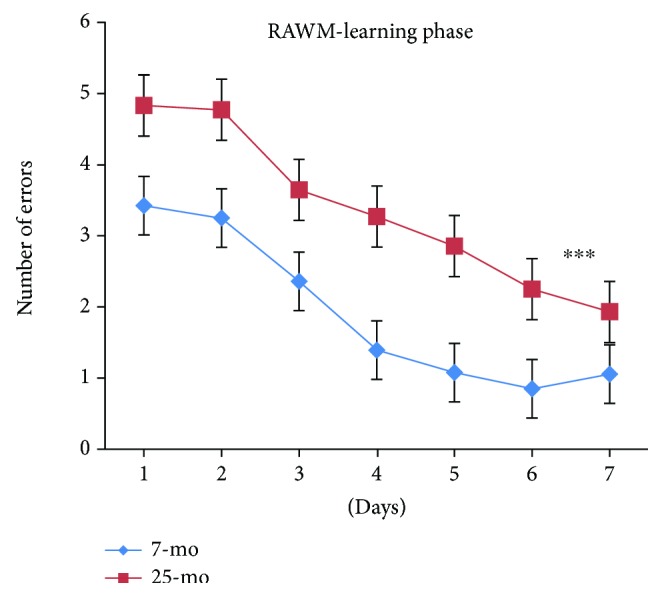
Number of errors of 7- and 25-month-old C57BL/6 mice in learning phase of RAWM (mean ± SEM). ^∗∗∗^*P* < 0.001.

**Figure 5:**
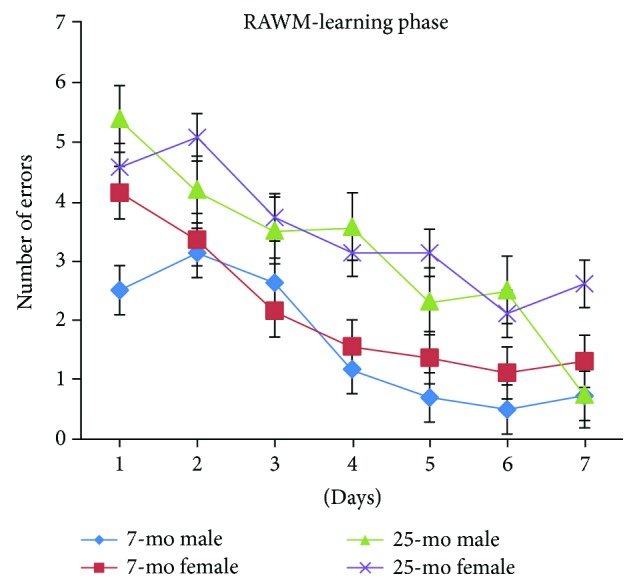
Number of errors of 7- and 25-month-old C57BL/6 male and female mice in learning phase of RAWM (mean ± SEM).

Overall, the number of errors committed while finding the submerged platform progressively and significantly decreased with day [*F*_(6,180)_ = 28.375, *P* < 0.001] (Figure 4). For mice overall, the number of errors significantly decreased by day 3 compared to error numbers on day 1 (*P* < 0.01) or day 2 (*P* < 0.05) and error numbers still significantly decreased on days 5 and 6 compared to numbers on day 4 (*P* < 0.05). Errors continued to reach significantly decreased levels on day 7 relative to day 5 (*P* < 0.05). In aged mice, the number of errors significantly increased, mirroring significant decline in spatial learning performance observed during the RAWM learning phase relative to performance of nonaged adult mice [*F*_(1,34)_ = 38.01, ^∗∗∗^*P* < 0.001] (Figure 4). Nonaged adult mice exhibited a decreasing trend in error numbers, with an average on day 4 of only one error, while aged mice exhibited an average of two errors at day 7. No significant gender-based differences were observed in error numbers, reflecting absence of gender influences on spatial learning performance during the RAWM learning phase for either aged or nonaged adult mice (*Ps* > 0.05) (Figure 5).

**Figure 6:**
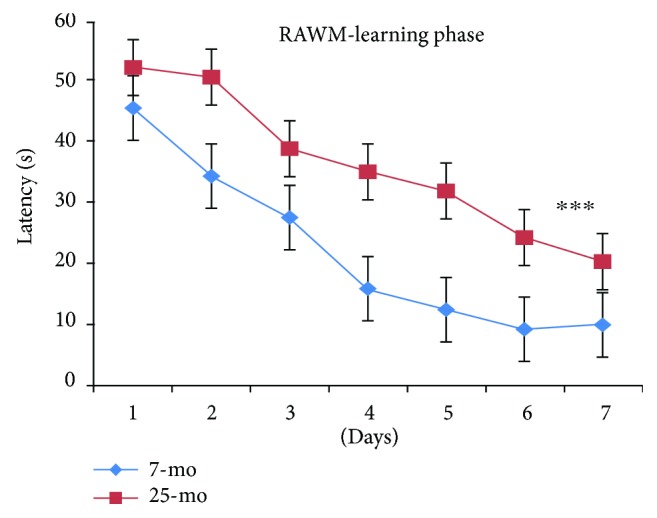
Latencies of 7- and 25-month-old C57BL/6 mice in learning phase of RAWM (s, mean ± SEM). ^∗∗∗^*P* < 0.001.

**Figure 7:**
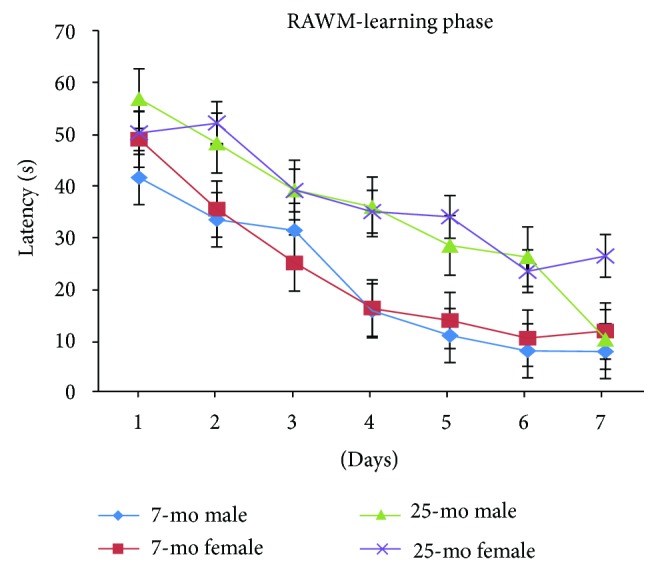
Latencies of 7- and 25-month-old C57BL/6 male and female mice in learning phase of RAWM (s, mean ± SEM).

As observed for errors, latencies for all mice progressively and significantly decreased with day [*F*_(6,180)_ = 51.753, *P* < 0.001] (Figure 6) and significantly decreasing on day 2 versus day 1 (*P* < 0.01) and even more significantly by day 3 relative to day 1 (*P* < 0.001) or day 2 (*P* < 0.01); latencies significantly decreased on day 4 relative to day 1 (*P* < 0.001), day 2 (*P* < 0.001), or day 3 (*P* < 0.05) and continued to significantly decrease by day 7 relative to latencies on day 4 (*P* < 0.001), day 5 (*P* < 0.001), or day 6 (*P* < 0.05). Latencies during the RAWM learning phase in aged mice were significantly higher than for nonaged adult mice, indicating decline in spatial learning ability with old age [*F*_(1,34)_ = 38.413, ^∗∗∗^*P* < 0.001] (Figure 6), but no significant gender-based latency differences were observed (*Ps* > 0.05) (Figure 7). Thus differences in spatial learning performance during the RAWM learning phase were observed between aged mice relative to nonaged adult mice, but no gender-based differences were observed. Finally, no increased floating tendency was observed in aged mice relative to nonaged mice during the RAWM learning phase.

### 3.4. Errors and Latencies during the RAWM Memory Phase

**Figure 8:**
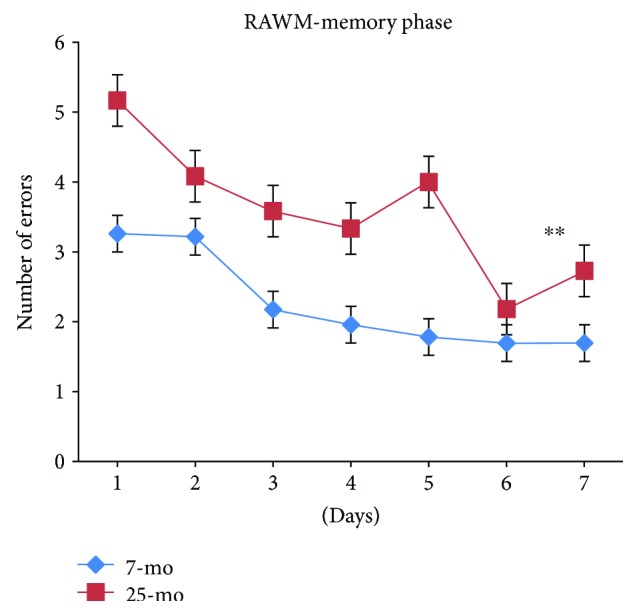
Number of errors of 7- and 25-month-old C57BL/6 mice in memory phase of RAWM (mean ± SEM). ^∗∗^*P* < 0.01.

**Figure 9:**
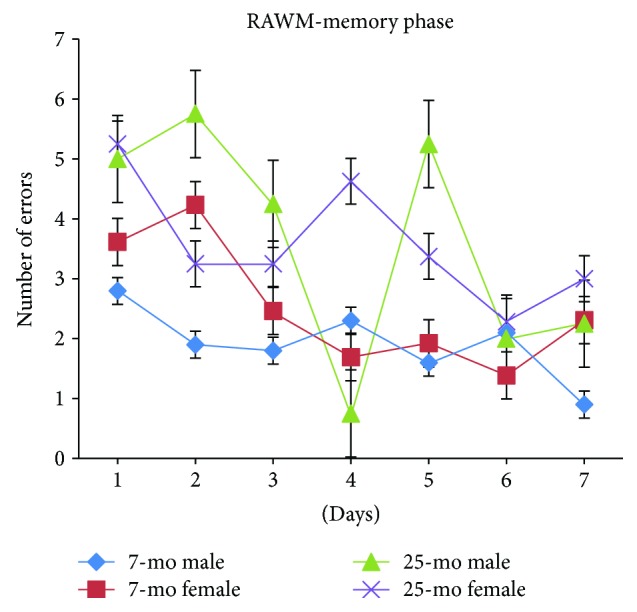
Number of errors of 7- and 25-month-old C57BL/6 male and female mice in memory phase of RAWM (mean ± SEM).

For all mice, numbers of errors committed while searching for the submerged platform progressively and significantly decreased with day [*F*_(6,180)_ = 4.461, *P* < 0.001] (Figure 8) and no significant differences in the number of errors on day 2 or day 3 were observed compared to error numbers on day 1 (*Ps* > 0.05). Numbers of errors continued to significantly decrease by day 4 compared to numbers on day 1 or 2 (*P* < 0.01 or *P* < 0.05), reaching significantly decreased levels on day 4 or 6 compared to those on day 2 (*P* < 0.05 or *P* < 0.01). Numbers of errors on day 7 were similar to those on day 5 or 6 (*Ps* > 0.05). For aged mice, numbers of errors significantly increased relative to the error numbers for nonaged adult mice, reflecting significant decline in spatial memory ability with advanced age as measured during the RAWM memory phase [*F*_(1,34)_ = 12.440, ^∗∗^*P* = 0.001 (<0.01)] (Figure 8). No significant gender-based effects were observed in the RAWM memory phase (*Ps* > 0.05) (Figure 9). In Figure 9, the 25-month-old male mice show a dip in error number at day 4 and unstable spatial memory performance.

**Figure 10:**
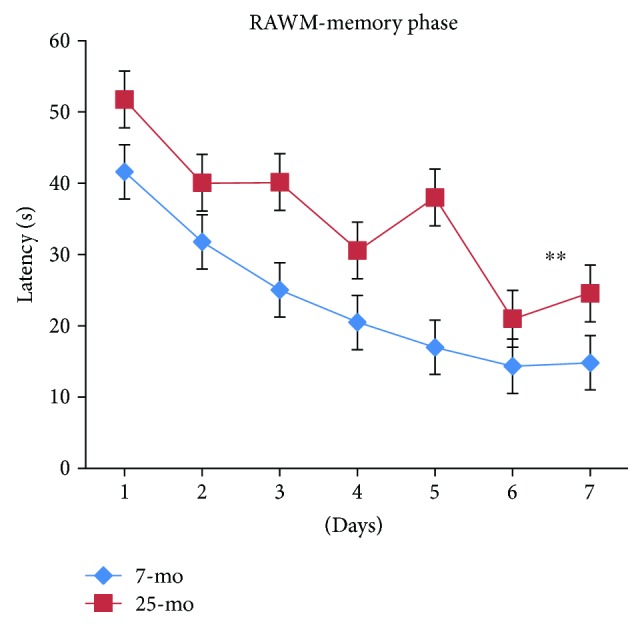
Latencies of 7- and 25-month-old C57BL/6 mice in memory phase of RAWM (s, mean ± SEM). ^∗∗^*P* < 0.01.

**Figure 11:**
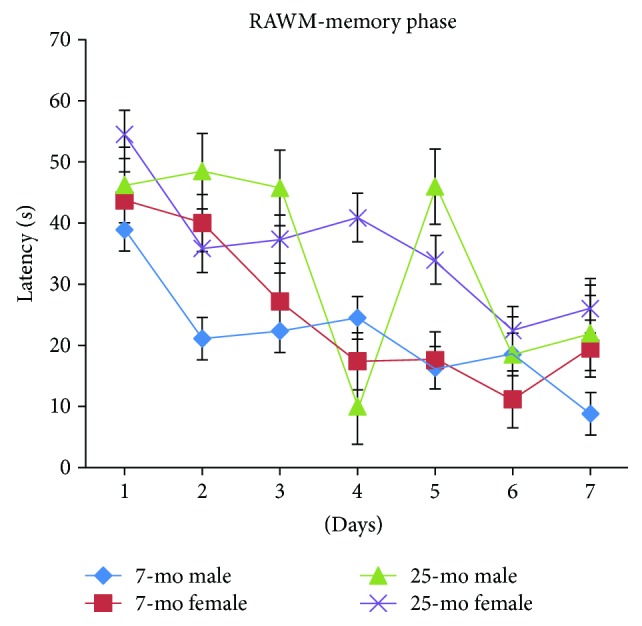
Latencies of 7- and 25-month-old C57BL/6 male and female mice in memory phase of RAWM (s, mean ± SEM).

For all mice, latencies progressively and significantly decreased with day [*F*_(6,180)_ = 9.968, *P* < 0.001] (Figure 10) and were similar on day 1 to day 2 (*P* > 0.05) and on days 3 to 5 (*P* > 0.05). Latencies on days 3 to 7 were significantly lower than latencies on day 1 (*P* < 0.05, *P* < 0.001, *P* < 0.01, *P* < 0.001, and *P* < 0.001). Latencies significantly decreased on day 4 or day 6 relative to day 2 (*P* < 0.01 or *P* < 0.001), and latencies on day 3 or day 5 were similar to those on day 2 (*Ps* > 0.05). On day 7, latencies were significantly decreased relative to latencies on days 1, 2, 3, and 5 (*P* < 0.001, *P* < 0.01, *P* < 0.01, and *P* < 0.05) but were similar to those on day 4 or 6 (*Ps* > 0.05). Latencies significantly declined more during the RAWM memory phase in nonaged adult mice versus aged mice [*F*_(1,34)_ = 13.613, ^∗∗^*P* = 0.001 (<0.01)] (Figure 10). Overall, no significant gender differences were observed for latencies, reflecting the absence of gender differences in spatial memory performance during the RAWM memory phase regardless of age (*Ps* > 0.05) (Figure 11). Furthermore, no increased floating tendency was observed in aged mice versus nonaged adult mice during the RAWM memory phase. In Figure 11, the 25-month-old male mice show a dip in latency at day 4 and unstable spatial memory performance.

### 3.5. Correlation of Nest Building and RAWM

There were no significant correlations among nesting scores in nest building and number of errors and latencies in RAWM in aged and nonaged adult male or female C57BL/6 mice (*Ps* > 0.05).

## 4. Discussion

Age-based and gender-based behavioral differences in nest building and RAWM performance were evaluated for aged and nonaged adult C57BL/6 mice. Significant age and gender differences were observed in nest building, while only age effects were observed in RAWM performance. With regard to nesting behavior, within each age group, significant gender-based nesting score differences were observed and differences were more pronounced for the 25-month-old (aged) group than for the 7-month-old (nonaged adult) group.

In MWM cued learning tests, latencies due to differences in swimming speed, nonspatial learning ability, visual acuity, and motor or sensorimotor functions in aged mice were similar to latencies in nonaged adult mice. Notably, swimming speeds, nonspatial learning ability, visual acuity, and movement capabilities of aged mice did not decline relative to those characteristics for younger adults; therefore, the abovementioned results suggest that significantly increased RAWM latencies in aged C57BL/6 mice were due to significant declines in spatial learning and memory (short-term memory, such as working memory and reference memory) abilities.

Successful completion of nest building in mice depends on hippocampal integrity and an intact medial prefrontal cortex [1, 2]. Here, nest building and RAWM spatial learning and memory capabilities significantly declined in aging C57BL/6 mice. Such behavioral declines may be related to serological, histochemical, and/or morphological changes in specific brain regions. Morphological changes previously associated with aging include changes in hippocampal subregions [8], altered serotonergic, dopaminergic, or cholinergic innervation, changes in dendritic arborization, altered dendritic spines, or altered adult neurogenesis, any or all of which may be responsible for observed behavioral changes in aged C57BL/6 mice and memory impairment in elderly humans.

We explored age- and gender-related behavioral differences in nest building and RAWM performance between aged and nonaged adult C57BL/6 mice in order to evaluate C57BL/6 mice as an aging mouse model. The results of this study provide evidence and add novel data demonstrating behavioral differences in nest building and RAWM performance between aged and nonaged adult wild-type C57BL/6 mice.

## 5. Conclusions

In C57BL/6 mice, nest building significantly declined in aged mice relative to nonaged adult mice, with greater declines in male versus female aged mice. Therefore, nest building is useful for detecting age-based and gender-based behavioral decline in C57BL/6 mice. In addition, RAWM results reported here demonstrate that spatial learning and memory significantly declined in aged C57BL/6 mice relative to these capabilities in younger adult mice. However, no significant gender differences were detected by RAWM testing regardless of age. In conclusion, both nest building and RAWM tests are sensitive tests for evaluation of age-related behavioral decline using C57BL/6 mouse animal model. This animal model and behavioral tests described here should be useful to further investigate mechanisms responsible for human age-related memory decline and to evaluate treatments or other ways to maintain aging brain function.
